# Maternal and infant factors had a significant impact on birthweight and longitudinal growth in a South African birth cohort

**DOI:** 10.1111/apa.14015

**Published:** 2017-09-04

**Authors:** S Budree, DJ Stein, K Brittain, E Goddard, N Koen, W Barnett, L Myer, HJ Zar

**Affiliations:** ^1^ Department of Paediatrics and Child Health Red Cross War Memorial Children's Hospital Cape Town South Africa; ^2^ MRC Unit on Child and Adolescent Health University of Cape Town Cape Town South Africa; ^3^ Department of Psychiatry & Mental Health Groote Schuur Hospital University of Cape Town Cape Town South Africa; ^4^ South African Medical Research Council Unit on Risk and Resilience in Mental Disorders Cape Town South Africa; ^5^ Division of Epidemiology and Biostatistics School of Public Health and Family Medicine University of Cape Town Cape Town South Africa

**Keywords:** Birth cohort, Birthweight, Infant growth, Low‐resource countries, Smoking

## Abstract

**Aim:**

This birth cohort study investigated longitudinal infant growth and associated factors in a multiethnic population living in a low‐resource district surrounding the town of Paarl in South Africa.

**Methods:**

Between March 2012 and October 2014, all mothers attending their second trimester antenatal visit at Paarl Hospital were approached for enrolment. Mother–infant pairs were followed from birth until 12 months of age. Comprehensive socio‐demographic, nutritional and psychosocial data were collected at birth, two, six and 12 months. Infant anthropometry was analysed as z‐scores for weight and height. Linear regression was used to investigate predictors of birthweight, and linear mixed‐effects models were used to investigate predictors of infant growth.

**Results:**

Longitudinal anthropometric data from 792 infants were included: 53% were Black African, 47% were mixed race, and 15% were born preterm. Stunting occurred in 13% of infants at 12 months. Maternal height, antenatal alcohol and tobacco use, ethnicity and socioeconomic status were significant predictors of birthweight. In the adjusted mixed‐effects model, birthweight was a significant predictor of growth during the first year of life.

**Conclusion:**

Birthweight was an important predictor of growth trajectory during infancy. Birthweight and growth were influenced by several important modifiable factors.

**Abbreviations**
AGA: Appropriate for gestational age
BMI: Body mass index
BMIZ: Body mass index for age z score
HFAZ: Height‐for‐age z‐score
IPV: Intimate partner violence
IQR: Interquartile range
LBW: Low birthweight
LMIC: Low and middle‐income countries
SES: Socioeconomic score
SGA: Small for gestational age
WFAZ: Weight‐for‐age z‐score
WFHZ: Weight‐for‐height z‐score
WHO: World Health Organization

**Key notes**

- This large cohort study investigated the factors associated with birthweight and longitudinal infant growth in a resource‐poor setting.
- Birthweight was a major predictor of growth trajectory during infancy, after adjusting for socioeconomic status, ethnicity, prematurity and intra‐uterine growth restriction.
- Alcohol or tobacco consumption during pregnancy was high in this population and was associated with both poor birth outcomes and poor postnatal growth during infancy.

## Introduction

Childhood malnutrition, which encompasses both undernutrition and overweight or obesity, is highly prevalent in low‐ and middle‐income countries (LMIC) 1. Although global rates of stunting in children under five years of age have decreased over the past decade, the prevalence of stunting in sub‐Saharan Africa has continued to rise 1. Obesity rates in children and adults in LMIC have also been rising, and these are referred to as the double burden of malnutrition 1, 2.

South Africa comprises multiethnic populations, with varying socioeconomic circumstances, psychosocial factors, disease exposures and cultural practices. Differences in child growth patterns may occur in such population groups, and these reflect genetic or environmental factors or a combination of these 3, 4. The prevalence of psychosocial factors, such as maternal depression, intimate partner violence (IPV) and substance abuse – including alcohol and tobacco consumption – in LMIC is high and has been associated with poor infant outcomes, including low birthweight and poor infant growth 5.

Few cohort studies from LMIC have investigated child growth longitudinally, and no longitudinal studies of growth patterns in South African infants from different ethnic backgrounds have been carried out. The aim of this study was to describe patterns of growth and factors that had an impact on growth in South African infants enrolled in the Drakenstein Child Health Study.

## Patients and methods

The primary aim of the Drakenstein Child Health Study birth cohort was to investigate the epidemiology, risk factors, aetiology and long‐term impact of respiratory illness on child health, including investigations into growth and nutrition in early childhood 6.

### Study population

Pregnant women living in a resource‐poor area surrounding an agricultural town were enrolled during their second trimester of pregnancy at two primary healthcare facilities serving different ethnic communities: TC Newman serving a mixed race community and Mbekweni serving a Black African community. This means that the two clinics were synonymous with ethnicity. Women who were less than 18 years of age, those who were unable to provide informed consent or those who intended to leave the area within a year were excluded. All the infants that were born to mothers enrolled into the study were included in the analysis. All pregnant women who were enrolled into the study received standard antenatal care at a single district level hospital.

### Measures

Socio‐demographic and health information were obtained from questionnaires administered to the mothers during antenatal and postnatal study visits. Four socio‐demographic variables were used to generate a relative socioeconomic score (SES): education, employment, household income and assets. Participants were stratified into quartiles based on their SES score. These quartiles were labelled as low, low‐to‐moderate, moderate‐to‐high or high SES, to enable comparisons within the study population 7.

All births occurred at a single public hospital, Paarl hospital, which provides general hospital and emergency care for a population of more than 600 000 people living in a vast geographical area of approximately 22 500 square kilometres. It is based in a predominantly semirural region 60 kilometres from Cape Town in the Southern tip of South Africa.

Birthweight and length were measured at the hospital. Gestational age at birth was estimated based on an antenatal ultrasound carried out in the second trimester (68%), and if this was unavailable, then fundal height (28%) or maternal recall of the last menstrual period (4%) was used. Trained study staff collected serial anthropometric measurements on the infants at two, six and 12 months. The infant's length was measured in centimetres to the nearest completed 0.5 cm, and their weight was measured in kilograms to the nearest completed 10 g, according to standard operating procedures. Maternal height was measured to the nearest 0.1 cm at enrolment, using a wall‐mounted stadiometer. The birth anthropometrical measurements were conducted by trained labour ward staff, and the birthweights and lengths were obtained from the patients’ folders. When we compared the labour ward staff measurements of infant anthropometry with study staff measurements in a subgroup of patients, we found no significant differences.

Weight was measured, in the light or no clothing, using the TAN1584 digital platform scale (Tanita, IL, USA) and recumbent length using the seca210 length‐measuring mat (seca, Hamburg, Germany). The latter was performed on a firm surface by two staff members. The equipment was calibrated weekly. All anthropometric measurements were carried out twice for each child, with a third measurement if the difference between the first and second measurement differed by more than 0.5 cm for length or more than 0.5 kg for weight. Study staff underwent regular anthropometric training and assessment every three months, and this was conducted by the lead anthropometrist.

Four time points were used for the purposes of this analysis: birth, two, six and 12 months of age. A valid anthropometric measurement was within four weeks before or after that time point.

With regard to nutritional information, the infant feeding practice was categorised based on the World Health Organization's (WHO) Infant and Young Child feeding indicators, namely exclusive breastfeeding or bottle feeding, which included infant cows’ milk‐based formula with or without breastmilk or semisolids 8.

Validated questionnaires were administered to mothers antenatally and six weeks postpartum to assess psychosocial factors 5. The Edinburgh Postnatal Depression Rating Scale was used to assess maternal depression and a score of at least 13 was classified as probable depression 5. An IPV questionnaire was used to assess recent exposure to physical, emotional and sexual IPV 5. Exposure was categorised as above the threshold if a participant reported more than one incident of IPV during the previous year. The Alcohol, Smoking and Substance Involvement Screening test was used to assess maternal alcohol consumption based on a scoring system that was previously described 5. Tobacco smoking was measured using maternal urinary cotinine levels antenatally and postnatally using the IMMULITE 1000 Nicotine Metabolite Kit (Siemens Medical Solutions Diagnostics, Caernarfon, UK). A urinary cotinine level of ≥500 ng/mL was used as the threshold to dichotomise mothers into active smokers and nonsmokers based on the manufacturer's recommendations.

### Statistical analysis

Birthweight and length were converted to z‐scores for gender and gestational age using the INTERGROWTH‐21st standards 9. Infants were classified into low birthweight (LBW) if they weighed less than 2500 g at birth, preterm if they born at less than 37 weeks of gestational age and small for gestational age (SGA) if they weighted less than the 10th percentile for gestational age, based on gender and gestational age. Corrected gestational age was used to calculate the WHO z‐score of preterm infants.

The weight and length measurements following birth were converted to z‐scores based on age and gender, using Anthro software (WHO, Geneva, Switzerland) 10. Weight‐for‐age z‐scores (WFAZ), height‐for‐age z‐scores (HFAZ), weight‐for‐height z‐scores (WFHZ) and body mass index (BMI) for age z scores (BMIZ) were calculated. BMI was calculated using the formula weight in kilograms divided by length in centimetres squared.

Growth patterns in the study population were analysed at each time point by calculating the median WFAZ and HFAZ as well as between the time points by calculating the difference in WFAZ and HFAZ. At each time point, indicators of malnutrition were calculated so that infants with an HFAZ <−2 were classified as stunted, those with a WFAZ <−2 as underweight for age, those with a WFHZ <−2 as wasted and those with a BMI for age >2 as overweight and >3 as obese. Growth patterns and indicators of malnutrition were stratified by study site, in other words ethnicity. Significant differences in WFAZ and HFAZ, indicators of malnutrition, and changes in WFAZ and HFAZ between time points were identified using chi‐square or Fisher's exact tests for categorical variables and *t*‐tests or Wilcoxon rank‐sum tests (Mann–Whitney tests) for continuous variables.

Variables significantly associated with infant WFAZ at birth were explored. Two linear regression models were built to investigate maternal height and antenatal smoking, based on urinary cotinine levels, as independent predictors of *a priori* interest. For all models, regression coefficients with 95% confidence intervals (95% CIs) were calculated to determine the strength of these associations.

Based on the literature, known variables associated with infant growth during the first year of life were explored using linear mixed‐effects models. Ethnicity and SES were included as fixed effects and WFAZ at birth and age at time of study visit were included as random effects. Three models were built. The first adjusted model (model one) included all variables that were significant in the unadjusted model. The final model (model two) only included the variables that remained significant in model one.

The statistical analysis was conducted using Stata 12 (StataCorp Inc, College Station, TX, USA).

Ethical approval was obtained from the University of Cape Town, Faculty of Health Sciences Human Research Ethics Committee and the Provincial Child Health Research committee. Written informed consent was obtained from all the participants.

## Results

From March 2012 to October 2014, we enrolled 789 mothers with a median age of 25.7 (21.8–30.7) years who had given birth to 792 infants, including three pairs of twins (Table 1) and all of these infants completed their birth study visit. At the time of analysis, 681 infants (93%) completed their two‐month study visit, 517 (71%) completed their six‐month study visit and 342 (47%) completed their 12‐month study visit (Fig. S1). We excluded 62 infants from the growth analysis due to incomplete data: 35 infants at two months and 27 infants at six months. In addition, 97 mother–infants pairs were lost to follow‐up during the course of the study. The mothers who were lost to follow‐up were significantly younger than those who remained in the study (median age 23.3 years versus 26.0 years; p = 0.003), had higher levels of education (p = 0.030) and were more likely to be primigravid (p = 0.048). There were no significant differences in birth characteristics between the infants who were lost to follow‐up and those who remained.

**Table 1:** Maternal demographic and psychosocial characteristics and infant birth outcomes

Variable	Mbekweni (Black African) – n (%)	TC Newman (Mixed race) – n (%)	Total – n (%)	p‐value
Maternal demographic and psychosocial characteristics				
Number of mothers	418 (53)	371 (47)	789	
Median [IQR] gestation at enrolment	23 [20‐26]	21 [19‐25]	22 [20‐25]	<0.001
Ethnicity				
Black/African	414 (99)	5 (1)	419 (53)	
Mixed race	4 (1)	363 (98)	367 (47)	
Other	0 (0)	3 (0.8)	3 (0.4)	
Median [IQR] age at enrolment (yrs)	26.7 [22.2‐31.8]	24.8 [21.3‐29.1]	25.7 [21.8‐30.7]	<0.001
Median [IQR] height (cm)	160 [156‐164]	158 [153‐162]	159 [155‐163]	<0.001
Educational achievement				
Primary education only	42 (10)	29 (8)	71 (9)	
Some secondary education	225 (54)	182 (49)	407 (52)	0.021
Completed secondary education	122 (29)	144 (39)	266 (34)	
Tertiary education	29 (7)	16 (4)	45 (6)	
Antenatal urine cotinine – active smoker	58 (14)	190 (51)	248 (31)	<0.001
Antenatal self‐reported alcohol use	34 (8)	105 (28)	139 (18)	<0.001
Antenatal depression – above threshold (EPDS)	114 (27)	90 (24)	204 (26)	0.335
Recent intimate partner violence (antenatal) – above threshold	110 (26)	146 (39)	256 (32)	<0.001
Primigravida	134 (32)	146 (39)	280 (35)	0.033
HIV‐infected	158 (38)	10 (3)	168 (21)	<0.001
Unemployed	327 (78)	260 (70)	587 (74)	0.009
Average household income				
<R1000/month	203 (49)	127 (34)	330 (42)	
R1000‐R5000/month	167 (40)	174 (47)	341 (43)	<0.001
>R5000/month	48 (11)	70 (19)	118 (15)	
SES quartile				
Lowest SES	144 (34)	69 (19)	213 (27)	<0.001
Low‐moderate SES	109 (26)	88 (24)	197 (25)	
Moderate‐high SES	99 (24)	94 (25)	193 (24)	
Highest SES	66 (16)	120 (32)	186 (24)	
Early postpartum maternal psychosocial characteristics (n = 366)				
Self‐reported tobacco use	6 (3)	91 (54)	97 (27)	<0.001
Self‐reported alcohol use	10 (5)	44 (26)	54 (15)	<0.001
Depression – above threshold (EPDS)	47 (24)	29 (17)	76 (21)	0.115
Recent intimate partner violence – above threshold	42 (21)	47 (28)	89 (24)	0.149
Infant birth outcomes				
Number of infants	421 (53)	371 (47)	792	
Infant sex				
Female	210 (50)	164 (44)	374 (47)	
Median [IQR] gestation at delivery (weeks)	39 [38‐40]	39 [37‐40]	39 [38‐40]	0.360
Preterm birth (<37 weeks)	64 (15)	55 (15)	119 (15)	0.882
Low birthweight (<2500 g)	45 (11)	71 (19)	116 (15)	0.001
Small for gestational age	96 (23)	107 (29)	203 (26)	0.052

The study population had a predominantly low income, with 43% of households earning below the minimum wage of 5000 South African Rands per month 11. Only half of the participants in the highest SES quartile reported a household income that was higher than that level. Just over a third (34%) of the Black African households fell into the lowest SES quartile and they were relatively poorer than mixed race households (Table 1). The reported levels of antenatal substance use were significantly higher in mothers of mixed race infants than Black African infants. More than half of the mixed race mothers were active smokers (51%) based on their antenatal urinary cotinine levels and more than a quarter (28%) reported consuming alcohol antenatally and postnatally. More than a quarter of mothers (26%) were classified as experiencing probable postnatal depression. Recent IPV was significantly higher among mixed race mothers than Black African mothers.

The median gestational age at birth was 39 weeks, but 15% of babies were born preterm (Table 1). More than a quarter of infants (26%) were born SGA while 15% were LBW (Table 1). The median WFAZ at birth was below the WHO reference population, and this was more marked in mixed race babies (Table 2). Of the 119 infants born preterm, 87 were late preterm (34–37 weeks), 20 were moderately preterm (32–34 weeks), and 12 were very preterm (<32 weeks). The median (IQR) gestation of the preterm infants was 35 (33–36) weeks. The median (IQR) birthweight of the preterm infants was 2.5 (2.05–2.88) kg. In total, 106 of the preterm infants were appropriate for gestational age (AGA), and 13 were SGA. The median WFAZ improved from birth to two months, with a median change of 0.2 between birth and two months. Between two and six months, infants exhibited higher weight gain than height gain. At six months, Black African infants were, on average, heavier and taller than mixed race infants. The period of highest weight gain during infancy was between birth and six months, with a difference in WFAZ of 0.7 (Table 2). At 12 months, the median WFAZ of the sample was almost identical to the WHO median, with a slowing up of weight gain between six and 12 months. Median HFAZ remained negative at all time points, indicating that the infants had slower linear growth and remained shorter than infants from the reference WHO population (Table 2). On average, children born to Black African mothers weighed more and were taller at birth and remained heavier and taller at each time point than those born to mixed race mothers (Table 2).

**Table 2:** Infant anthropometry and feeding

Variable	Mbekweni (Black African)	TC Newman (Mixed race)	Total	p‐value
Birth, (n)	421	371	792	
Median WFAZ [IQR]	−0.4 [−1.2 to 0.3]	−0.7 [−1.4 to −0.1]	−0.5 [−1.3 to 0.1]	<0.001
Median HFAZ [IQR]	0.1 [−0.8 to 1.1]	0.003 [0.8 to 0.9]	0.1 [−0.8 to 1.0]	0.019
2 months, (n)	344	337	681	
Median WFAZ [IQR]	−0.1 [−0.9 to 0.6]	−0.5 [−1.4 to 0.2]	−0.3 [−1.1 to 0.4]	<0.001
Median HFAZ [IQR]	−0.5 [−1.3 to 0.5]	−0.9 [−1.9 to 0.0]	−0.7 [−1.6 to 0.2]	<0.001
Median change in WFAZ between birth and 2 months [IQR] (n* *=* *681)	0.3 [−0.5 to 1.0]	0.1 [−0.5 to 0.7]	0.2 [−0.5 to 0.9]	0.017
Median change in HFAZ between birth and 2 months [IQR] (n* *=* *681)	−0.7 [−1.6 to 0.3]	−1.0 [−1.8 to 0.1]	−0.9 [−1.7 to 0.2]	0.040
6 months, (n)	243	274	517	
Median WFAZ [IQR]	0.5 [−0.4 to 1.2]	−0.05 [−1.0 to 0.8]	0.2 [−0.7 to 1.0]	<0.001
Median HFAZ [IQR]	−0.2 [−1.3 to 0.7]	−0.7 [−1.6 to 0.2]	−0.4 [−1.5 to 0.5]	0.002
Median change in WFAZ between 2 and 6 months [IQR] (n* *=* *500)	0.5 [−0.1 to 1.2]	0.4 [−0.1 to 1.0]	0.4 [−0.1 to 1.1]	0.455
Median change in HFAZ between 2 and 6 months [IQR] (n* *=* *500)	0.3 [−0.8 to 1.3]	0.2 [−0.5 to 0.9]	0.2 [−0.6 to 1.1]	0.732
12 months, (n)	155	187	342	
Median WFAZ [IQR]	0.3 [−0.5 to 1.0]	−0.1 [−1.1 to 0.6]	0.1 [−0.8 to 0.8]	<0.001
Median HFAZ [IQR]	−0.3 [−1.2 to 0.6]	−0.6 [−1.6 to 0.2]	−0.5 [−1.4 to 0.4]	0.014
Median change in WFAZ between 6 and 12 months [IQR] (n* *=* *305)	0.1 [−0.5 to 0.6]	−0.03 [−0.5 to 0.3]	0.02 [−0.5 to 0.4]	0.262
Median change in HFAZ between 6 and 12 months [IQR] (n* *=* *305)	−0.01 [−0.9 to 0.8]	0.2 [−0.7 to 0.8]	0.1 [−0.8 to 0.8]	0.417
Infant feeding categories				
2 months, (n)	344	337	681	
Exclusive breastfeeding	143 (42)	163 (48)	306 (45)	
Exclusive formula feeding	81 (24)	11 (3)	92 (14)	<0.001
Mixed feeding	120 (35)	163 (48)	283 (42)	
6 months, (n)	243	274	517	
Exclusive breastfeeding	22 (9)	23 (8)	45 (9)	
Exclusive formula feeding	31 (13)	3 (1)	34 (7)	<0.001
Mixed feeding	190 (78)	248 (91)	438 (85)	
12 months, (n)	155	187	342	
Mixed breastfeeding	57 (37)	117 (63)	177 (51)	
Mixed formula feeding	91 (59)	53 (28)	149 (43)	<0.001
Solids only	7 (5)	17 (9)	24 (7)	

WFAZ, weight‐for‐age z‐score; HFAZ, height‐for‐age z‐score.

Malnutrition, either under or over nutrition, occurred consistently throughout infancy (Table 3). Stunting was the most prevalent indicator of undernutrition at all time points, ranging from 17% of infants at two months to 13% by 12 months. At 12 months of age, 2% of the children were obese and 6% were overweight and the rates were higher among black African than mixed race infants (Table 3).

**Table 3:** Prevalence of infant malnutrition

Variable	Mbekweni (Black African) – n (%)	TC Newman (Mixed race) – n (%)	Total – n (%)	p‐value
2 months, (n)	344	337	681	
Wasting	22 (6)	9 (3)	31 (5)	0.020
Stunting	42 (12)	73 (22)	115 (17)	0.001
Underweight for age	20 (6)	39 (12)	59 (9)	0.008
Overweight	18 (5)	10 (3)	28 (4)	0.137
Obese	4 (1)	1 (0.3)	5 (0.7)	0.186
6 months, (n)	243	274	517	
Wasting	13 (5)	8 (3)	21 (4)	0.162
Stunting	25 (10)	45 (16)	70 (14)	0.042
Underweight for age	7 (3)	21 (8)	28 (5)	0.016
Overweight	31 (13)	16 (6)	47 (9)	0.006
Obese	17 (7)	8 (3)	25 (5)	0.031
12 months, (n)	155	187	342	
Wasting	3 (2)	9 (5)	12 (4)	0.237
Stunting	12 (8)	31 (17)	43 (13)	0.014
Underweight for age	3 (2)	16 (9)	19 (6)	0.008
Overweight	14 (9)	8 (4)	22 (6)	0.074
Obese	6 (4)	1 (0.5)	7 (2)	0.030

In the unadjusted models of infant WFAZ at birth, ethnicity, household income, gravidity, maternal alcohol and tobacco use, recent IPV and maternal height were significantly associated with lower WFAZ at birth (Table 4). Only variables that were significant in the unadjusted models were included into the multivariate model. Psychosocial and nutritional models were built separately. In the psychosocial multivariate model, the associations between both maternal alcohol and tobacco use and lower WFAZ at birth remained significant, independent of ethnicity and SES (Table 4). In the nutritional model, increased maternal height, which was used as a proxy for maternal nutritional status, remained significantly associated with higher infant WFAZ at birth, when adjusted for ethnicity and SES (Table 4).

**Table 4:** Association between maternal factors and infant weight‐for‐age z‐score (WFAZ) at birth

Variable	Median WFAZ [IQR]	Unadjusted regression coefficient [95% CI]	p‐value	(A) Psychosocial‐behavioural model: Adjusted regression coefficient [95% CI]	p‐value	(B) Nutritional model: Adjusted regression coefficient [95% CI]	p‐value
Recruitment site							
TC Newman (Mixed race)	−0.7 [−1.4 to −0.1]	Reference		Reference		Reference	
Mbekweni (Black African)	−0.4 [−1.2 to 0.3]	0.3 [0.2 to 0.5]	<0.001	0.2 [0.01 to 0.3]	0.040	0.3 [0.1 to 0.4]	<0.001
SES quartile							
Highest SES	−0.4 [−1.0 to 0.3]	Reference		Reference		Reference	
Moderate‐high SES	−0.7 [−1.4 to 0.01]	−0.2 [−0.4 to −0.01]	0.038	−0.2 [−0.4 to −0.03]	0.027	−0.3 [−0.5 to −0.1]	0.011
Low‐moderate SES	−0.5 [−1.4 to 0.04]	−0.2 [−0.4 to −0.01]	0.044	−0.2 [−0.4 to 0.001]	0.051	−0.2 [−0.5 to −0.04]	0.019
Lowest SES	−0.7 [−1.4 to 0.04]	−0.2 [−0.4 to 0.01]	0.068	−0.2 [−0.4 to −0.003]	0.046	−0.3 [−0.5 to −0.05]	0.016
Gravidity							
Multigravida	−0.4 [−1.2 to 0.2]	Reference					
Primigravida	−0.8 [−1.5 to −0.1]	−0.4 [−0.5 to −0.2]	<0.001				
Antenatal urine cotinine							
Nonsmoker	−0.4 [−1.1 to 0.3]	Reference		Reference			
Active smoker	−0.8 [−1.5 to −0.2]	−0.4 [−0.6 to −0.3]	<0.001	−0.3 [−0.5 to −0.1]	0.001		
Antenatal alcohol use							
No self‐reported alcohol use	−0.5 [−1.2 to 0.2]	Reference		Reference			
Any self‐reported alcohol use	−0.9 [−1.6 to −0.3]	−0.4 [−0.6 to −0.2]	<0.001	−0.3 [−0.5 to −0.1]	0.010		
Antenatal depression (EPDS)							
Below threshold	−0.5 [−1.3 to 0.1]	Reference					
Above threshold	−0.6 [−1.3 to −0.01]	−0.1 [−0.2 to 0.1]	0.554				
Recent intimate partner violence							
Below threshold	−0.5 [−1.2 to 0.1]	Reference					
Above threshold	−0.6 [−1.4 to 0.1]	−0.2 [−0.3 to −0.003]	0.045				
Maternal height		0.03 [0.02 to 0.04]	<0.001			0.02 [0.01 to 0.03]	<0.001

In the univariate analysis, birthweight, ethnicity, SES, antenatal and postnatal IPV and antenatal and postnatal maternal substance use – specifically alcohol or tobacco – were significantly associated with infant growth, as measured by repeated WFAZ measurements over the first year of life (Table 5). However, only birthweight and antenatal and postnatal maternal substance use remained significantly associated with infant growth after controlling for ethnicity and SES. In the final multivariate model, birthweight, ethnicity, SES and antenatal maternal substance use remained significantly associated with growth during infancy, adjusted for infant age at the time of measurement. Infant WFAZ at birth was an important predictor of WFAZ throughout the first year, independent of ethnicity, SES and antenatal maternal substance use.

**Table 5:** Mixed‐effects model of variables associated with infant weight‐for‐age z‐scores (WFAZ; repeated measurements) at two months, six months, and 12 months

Variable	Unadjusted regression coefficient [95% CI]	p‐value	Model 1		Model 2	
			Adjusted regression coefficient [95% CI]^a^	p‐value	Adjusted regression coefficient [95% CI]^b^	p‐value
Clinic (n* *=* *704)						
TC Newman (Mixed Race)	Reference		Reference		Reference	
Mbekweni (Black African)	0.5 [0.3 to 0.6]	<0.001	0.6 [0.4 to 0.8]	<0.001	0.3 [0.1 to 0.4]	<0.001
SES quartile (n* *=* *704)						
Highest SES	Reference		Reference		Reference	
Moderate‐high SES	−0.2 [−0.4 to 0.02]	0.072	−0.3 [−0.5 to −0.1]	0.009	−0.1 [−0.3 to 0.1]	0.531
Low‐moderate SES	−0.3 [−0.5 to −0.02]	0.031	−0.4 [−0.6 to −0.2]	0.001	−0.1 [−0.3 to 0.06]	0.171
Lowest SES	−0.6 [−0.8 to −0.3]	<0.001	−0.8 [−1.0 to −0.5]	<0.001	−0.5 [−0.7 to −0.3]	<0.001
WfA z‐score at birth (n* *=* *704)	0.5 [0.5 to 0.6]	<0.001	0.5 [0.4 to 0.6]	<0.001	0.5 [0.4 to 0.6]	<0.001
Recent intimate partner violence (antenatal; n* *=* *704)						
Below threshold	Reference		Reference			
Above threshold	−0.2 [−0.4 to −0.03]	0.021	−0.1 [−0.3− 0.1]	0.309		
Recent intimate partner violence (early postpartum; n* *=* *360)						
Below threshold	Reference		Reference			
Above threshold	−0.4 [−0.7 to −0.1]	0.010	−0.3 [−0.5− 0.001]	0.051		
Antenatal urine cotinine (n* *=* *704)						
Non‐smoker	Reference		Reference		Reference	
Active smoker	−0.6 [−0.8 to −0.4]	<0.001	−0.4 [−0.6 to −0.2]	<0.001	−0.2 [−0.4 to −0.04]	0.016
Early postpartum tobacco use (n* *=* *360)						
No self‐reported tobacco use	Reference		Reference			
Any self‐reported tobacco use	−0.5 [−0.8 to −0.3]	<0.001	−0.2 [−0.5 to −0.2]	0.338		
Antenatal alcohol use (n* *=* *704)						
No self‐reported alcohol use	Reference		Reference		Reference	
Any self‐reported alcohol use	−0.5 [−0.7 to −0.3]	<0.001	−0.4 [−0.6 to −0.1]	0.001	−0.2 [−0.4 to −0.02]	0.029
Early postpartum alcohol use (n* *=* *360)						
No self‐reported alcohol use	Reference		Reference			
Any self‐reported alcohol use	−0.2 [−0.6 to 0.1]	0.161	0.02 [−0.3 to 0.4]	0.913		

a Adjusted for recruitment site and SES quartile.

b Adjusted for all other covariates in model and for infant age at time of measurement.

## Discussion

In this South African birth cohort, we found a high prevalence of LBW and SGA infants and a mean WFAZ at birth below the WHO reference population. Despite some catch‐up growth, a substantial prevalence of childhood stunting was observed in this sample. Black African children had better nutritional status from birth that persisted throughout infancy compared to mixed race infants. We also demonstrated an emerging group of overweight and obese infants, with a higher prevalence among black African infants. Antenatal maternal smoking and alcohol consumption and maternal height were important independent predictors of infant birthweight. Birthweight, SES, ethnicity and maternal antenatal alcohol and tobacco consumption were important determinants of the infant's longitudinal growth during the first year of life.

Our results were consistent with studies that showed that birthweight was a strong determinant of longitudinal infant growth in the first year 1. In our sample, factors such as maternal smoking and alcohol consumption during pregnancy, gravidity and poor maternal nutrition were strong predictors of a lower weight at birth (Table 5) and were consistent with findings from other studies 1, 12, 13, 14. LBW is a significant risk factor for poor growth during infancy and may be due to prematurity, being born SGA or a combination of these factors 1. The prevalence of LBW infants in this study was higher than the figures reported by other African and South African studies 15, 16. Our findings that birthweight was a predictor of growth during infancy may be due to the high prevalence of LBW and SGA infants in this population. However infant birthweight was still a significant independent predictor of infant growth during the first year in the subgroup analysis, when infants with LBW or SGA were excluded (p < 0.001) (Table S1). As demonstrated in other studies, infants born term SGA and preterm SGA had significantly slower growth than those born preterm and term AGA (Table S2, Fig. S2) 1, 17, 18. Only 10% of the preterm infants were early preterm, which is a very small proportion.

We demonstrated the independent negative effects that smoking and alcohol consumption during pregnancy had on both birthweight and postnatal infant growth, which persisted after controlling for SES and ethnicity. This may have been partially mediated through the effects of alcohol and tobacco on foetal growth 14, 19. However, further investigations into the mechanistic effects on postnatal growth are required, for example the effects of these substances on infant micronutrient status, foetal programming, the intestinal microbiome and epigenetic alterations 20. Similar effects of antenatal tobacco or alcohol exposure on infant growth have been shown in other studies 14, 21.

Socioeconomic score (SES) was an important determinant of infant growth. Consistent with other studies in LMIC, higher SES promoted infant growth 22. This may have been due to better maternal health, better nutritional knowledge, being more able to afford formula or complementary feeding and better health seeking behaviour 23. Ethnic disparities in infant health have been described in several multiethnic countries globally and have mainly resulted from differences in maternal health, access to healthcare services and SES 4. Unexpectedly, black African infants had higher birthweights and better longitudinal growth than mixed race infants, despite coming from lower SES households, and these effects persisted after controlling for birthweight and SES. Additional genetic or environmental interactions may be important in determining growth during infancy and these require further investigation 1.

We found a high prevalence of stunting in this sample, particularly at two months of age, with a marginal decline by 12 months of age. These results were consistent with cross‐sectional South African studies that have reported a similar high prevalence of stunting in children 24. The high prevalence of stunting is surprising given the study setting, with access to healthcare facilities, free maternal antenatal care, nutritional programmes and a strong primary healthcare programme. SES, maternal, nutritional, genetic, or infant factors, such as environmental enteric dysfunction may have been contributory factors and further studies of these are underway.

In contrast, we also found a concerning prevalence of overweight or obese infants. To the best of our knowledge, no previous studies have documented the prevalence of overweight and obese infants in South Africa. These findings may reflect changes in lifestyle, diet or poor feeding practices 25 and a detailed analysis of such factors is currently being undertaken. A previous study within this population showed no correlation between birthweight and overweight or obesity at 12 months of age 26.

The strengths of this study included the prospective design with good cohort retention, the longitudinal measurements of multiple parameters of infant growth by trained study staff, the inclusion of two ethnically distinct South African population groups and the collection of comprehensive SES, maternal and nutritional information. In addition, using mixed‐effects modelling of growth enabled us to model individual growth trajectories, which are by nature comprised of repeated, dependent measurements over time, in a single model. This added to the quality of our results. Some limitations also need to be noted and these were that these findings may not be generalisable to other African or LMIC populations. However, many of the factors explored in this analysis are highly prevalent in LMIC.

This was the first study of this magnitude to evaluate longitudinal growth in a developing county during the first year of life, including multiple maternal, infant, psychosocial and environmental risk factors that have been associated with poor infant growth. It highlights some of the adversities that children in developing countries face, which arise as early as intra‐uterine life.

## Conclusion

Our findings of the predictive nature of birthweight on growth trajectory during infancy emphasise the importance of maternal health and antenatal care on infant growth. Simple, cost‐effective interventions that focus on maternal health, such as programmes to reduce alcohol consumption and smoking and improve nutrition, may improve birthweights and subsequent postnatal growth, thereby promoting child health. Further studies are required to investigate environmental and genetic determinants of infant growth in LMIC.

## Financial support

The Drakenstein Child Health Study is funded by the Bill and Melinda Gates Foundation (OPP 1017641), The Discovery Foundation, South Africa, The South African Medical Research Council, GlaxoSmithKline (GSK)/South African Thoracic Society, The Harry Crossley Foundation and The Hamilton‐Naki Foundation

## Conflict of interest

There authors have no conflicts of interest to declare.

## Supporting information


**Figure S1** Flow chart of infants included at each study visit between March 2012 and October 2014.
**Figure S2** Longitudinal infant WFA z scores stratified by prematurity and weight.
**Table S1** Mixed effects model of variables associated with infant weight‐for‐age z‐scores (WFAZ; repeated measurements) at 2 months, 6 months, and 12 months; infants born preterm and/or SGA excluded.
**Table S2** Mixed effects model of variables associated with infant weight‐for‐age z‐scores (WFAZ; repeated measurements) at 2 months, 6 months, and 12 months; restricted to infants born preterm and/or SGA.
