# Supplementary material for: Maternal and infant factors had a significant impact on birthweight and longitudinal growth in a South African birth cohort
Source: Acta Paediatr. 2017 Sep 4;106(11):1793–801. doi: 10.1111/apa.14015 (PMC5656834; doi:10.1111/apa.14015)
Supplement: Supplementary file 1 — Figure S1 Flow chart of infants included at each study visit between March 2012 and October 2014. Figure S2 Longitudinal infant WFA z scores stratified by prematurity and weight. Table S1 Mixed effects model of variables associated with infant weight‐for‐age z‐scores (WFAZ; repeated measurements) at 2 months, 6 months, and 12 months; infants born preterm and/or SGA excluded. Table S2 Mixed effects model of variables associated with infant weight‐for‐age z‐scores (WFAZ; repeated measurements) at 2 months, 6 months, and 12 months; restricted to infants born preterm and/or SGA. [file APA-106-1793-s001.docx]

Supplementary Material: Nutritional Status and Growth of Infants in a South African Birth Cohort


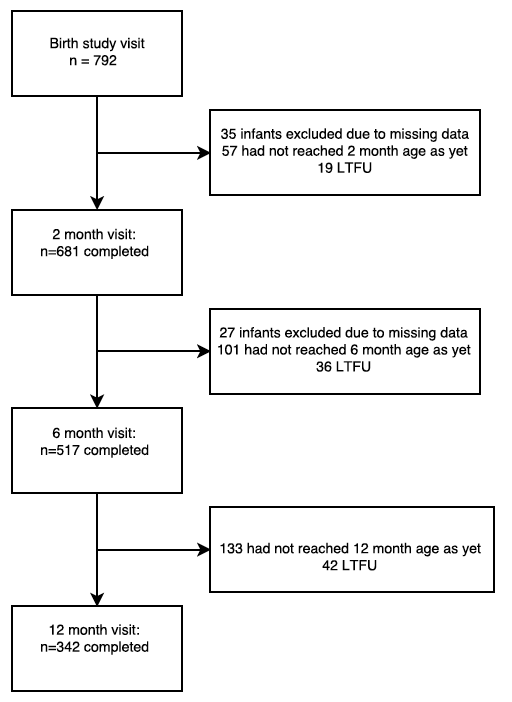


Figure S1: Flow chart of infants included at each study visit between March 2012 and October 2014

LTFU: loss to follow up

WFA Z score

Figure S2: Longitudinal infant WFA z scores stratified by prematurity and weight

| **Table S1** Mixed effects model of variables associated with infant weight-for-age z-scores (WFAZ; repeated measurements) at 2 months, 6 months, and 12 months; infants born preterm and/or SGA excluded | | | | | | |
| --- | --- | --- | --- | --- | --- | --- |
| Variable | Unadjusted regression coefficient  [95% CI] | *P*-value | **Model 1** | | **Model 2** | |
|  |  |  | Adjusted regression coefficient  [95% CI]^a^ | *P*-value | Adjusted regression coefficient  [95% CI]^b^ | *P*-value |
| Clinic (*n*=508)  TC Newman (Mixed Race)  Mbekweni (Black African) | Reference  0.3 [0.2, 0.5] | <0.001 | Reference  0.4 [0.3, 0.6] | <0.001 | Reference  0.2 [0.1, 0.4] | 0.003 |
| SES quartile (*n*=508)  Highest SES  Moderate-high SES  Low-moderate SES  Lowest SES | Reference  -0.2 [-0.4, 0.1]  -0.2 [-0.4, 0.1]  -0.5 [-0.7, -0.2] | 0.197  0.196  <0.001 | Reference  -0.2 [-0.5, 0.01]  -0.2 [-0.5, -0.01]  -0.6 [-0.8, -0.4] | 0.057  0.042  <0.001 | Reference  -0.1 [-0.3, 0.2]  -0.1 [-0.3, 0.1]  -0.3 [-0.5, -0.1] | 0.620  0.376  0.011 |
| WfA z-score at birth (*n*=508) | 0.5 [0.4, 0.6] | <0.001 | 0.5 [0.4, 0.6] | <0.001 | 0.5 [0.4, 0.6] | <0.001 |
| Recent intimate partner violence (antenatal; *n*=508)  Below threshold  Above threshold | Reference  -0.1 [-0.3, 0.1] | 0.420 | Reference  -0.02 [-0.2, 0.2] | 0.842 |  |  |
| Recent intimate partner violence (early postpartum; *n*=258)  Below threshold  Above threshold | Reference  -0.2 [-0.4, 0.1] | 0.297 | Reference  -0.1 [-0.4, 0.2] | 0.382 |  |  |
| Antenatal urine cotinine (*n*=508)  Non-smoker  Active smoker | Reference  -0.4 [-0.6, -0.2] | <0.001 | Reference  -0.3 [-0.5, -0.05] | 0.015 | Reference  -0.1 [-0.3, 0.1] | 0.312 |
| Early postpartum tobacco use (*n*=258)  No self-reported tobacco use  Any self-reported tobacco use | Reference  -0.3 [-0.6, -0.1] | 0.020 | Reference  0.01 [-0.4, 0.4] | 0.969 |  |  |
| Antenatal alcohol use (*n*=508)  No self-reported alcohol use  Any self-reported alcohol use | Reference  -0.5 [-0.7, -0.2] | <0.001 | Reference  -0.4 [-0.6, -0.1] | 0.001 | Reference  -0.2 [-0.4, -0.04] | 0.019 |
| Early postpartum alcohol use (*n*=258)  No self-reported alcohol use  Any self-reported alcohol use | Reference  -0.1 [-0.5, 0.2] | 0.455 | Reference  0.1 [-0.3, 0.5] | 0.657 |  |  |
| ^a^ Adjusted for recruitment site and SES quartile; ^b^ Adjusted for all other covariates in model and for infant age at time of measurement | | | | | | |

| **Table S2** Mixed effects model of variables associated with infant weight-for-age z-scores (WFAZ; repeated measurements) at 2 months, 6 months, and 12 months; restricted to infants born preterm and/or SGA | | | | | | |
| --- | --- | --- | --- | --- | --- | --- |
| Variable | Unadjusted regression coefficient  [95% CI] | *P*-value | **Model 1** | | **Model 2** | |
|  |  |  | Adjusted regression coefficient  [95% CI]^a^ | *P*-value | Adjusted regression coefficient  [95% CI]^b^ | *P*-value |
| Clinic (*n*=196)  TC Newman (Mixed Race)  Mbekweni (Black African) | Reference  0.8 [0.5, 1.1] | <0.001 | Reference  0.9 [0.6, 1.3] | <0.001 | Reference  0.3 [-0.002, 0.7] | 0.051 |
| SES quartile (*n*=196)  Highest SES  Moderate-high SES  Low-moderate SES  Lowest SES | Reference  -0.2 [-0.8, 0.4]  -0.3 [-0.8, 0.3]  -0.6 [-1.1, 0.004] | 0.518  0.360  0.052 | Reference  -0.3 [-0.9, 0.2]  -0.6 [-1.1, 0.01]  -0.9 [-1.4, -0.3] | 0.249  0.054  0.002 | Reference  0.01 [-0.4, 0.4]  -0.1 [-0.6, 0.3]  -0.7 [-1.1, -0.3] | 0.981  0.562  0.001 |
| WfA z-score at birth (*n*=196) | 0.5 [0.4, 0.6] | <0.001 | 0.5 [0.3, 0.6] | <0.001 | 0.5 [0.4, 0.6] | <0.001 |
| Recent intimate partner violence (antenatal; *n*=196)  Below threshold  Above threshold | Reference  -0.4 [-0.8, -0.04] | 0.029 | Reference  -0.1 [-0.5, 0.2] | 0.520 |  |  |
| Recent intimate partner violence (early postpartum; *n*=102)  Below threshold  Above threshold | Reference  -0.7 [-1.2, -0.2] | 0.012 | Reference  -0.4 [-1.0, 0.2] | 0.167 |  |  |
| Antenatal urine cotinine (*n*=196)  Non-smoker  Active smoker | Reference  -0.8 [-1.2, -0.5] | <0.001 | Reference  -0.5 [-0.8, -0.1] | 0.014 | Reference  -0.3 [-0.7, -0.03] | 0.031 |
| Early postpartum tobacco use (*n*=102)  No self-reported tobacco use  Any self-reported tobacco use | Reference  -0.8 [-1.3, -0.3] | 0.002 | Reference  -0.5 [-1.1, 0.1] | 0.093 |  |  |
| Antenatal alcohol use (*n*=196)  No self-reported alcohol use  Any self-reported alcohol use | Reference  -0.5 [-0.9, -0.1] | 0.011 | Reference  -0.2 [-0.6, 0.2] | 0.329 | Reference  0.1 [-0.3, 0.4] | 0.641 |
| Early postpartum alcohol use (*n*=102)  No self-reported alcohol use  Any self-reported alcohol use | Reference  -0.2 [-0.8, 0.4] | 0.557 | Reference  0.1 [-0.5, 0.7] | 0.735 |  |  |
| ^a^ Adjusted for recruitment site and SES quartile; ^b^ Adjusted for all other covariates in model and for infant age at time of measurement | | | | | | |
